# Effect of Triterpenoid Saponins as Foaming Agent on Mechanical Properties of Geopolymer Foam Concrete

**DOI:** 10.3390/ma17163921

**Published:** 2024-08-07

**Authors:** Xiaoyu Wang, Yangyang Wu, Xiangguo Li, Yuheng Li, Wen Tang, Jianming Dan, Chenglin Hong, Jinyu Wang, Xiaoqiang Yang

**Affiliations:** 1 School of Chemistry and Chemical Engineering, Key Laboratory for Green Processing of Chemical Engineering of Xinjiang Bingtuan, Shihezi University, Shihezi 832003, China; wxy020026@163.com (X.W.); m15999165967@163.com (Y.W.); 16643246889@163.com (Y.L.); tw1226999@163.com (W.T.); djm_tea@shzu.edu.cn (J.D.); hcl_tea@shzu.edu.cn (C.H.); 2 State Key Laboratory of Silicate Materials for Architectures, Wuhan University of Technology, Wuhan 430074, China; lxggroup@163.com; 3 Xinjiang Beixin Building Materials Industry Group Co., Ltd., Uygur 830011, China

**Keywords:** geopolymer, foam agents, triterpenoid saponin, pore structure, compressive strength, density, surfactant

## Abstract

Geopolymer foam concrete (GFC), an emerging thermal insulation material known for its environmentally friendly and low-carbon attributes, has gained prominence for its use in bolstering building energy efficiency. A critical challenge in GFC production is foam destabilization by the alkaline environment in which foam is supersaturated with salt. In this study, GFC was prepared by using triterpene saponin (TS), sodium dodecyl sulphate (SDS), and cetyltrimethylammonium bromide (CTAB) as blowing agents, with fly ash as the precursor and calcium carbide slag (CA) combined with Glauber’s salt (GS, Na_2_SO_4_ ≥ 99%) as the activator. The effect of GFC on mechanical properties was analyzed by examining its fluidity, pore structure, dry density, and compressive strength. The results show that TS has a stable liquid film capable of adapting to the adverse effects of salt supersaturation and alkaline environments. TS is highly stable in the GFC matrix, and so the corresponding pore size is small, and the connectivity is low in the hardened GFC. In addition, the hydration products of GFC exhibit different morphologies depending on the surfactant used. TS has better water retention due to hydrogen bonding, which facilitates the hydration process.

## 1. Introduction

Foam concrete is a composite material widely used in building envelopes. It is known for its commendable mechanical properties, low thermal conductivity, and fire-resistant attributes [1,2,3]. Preparing foam concrete requires a large amount of cement; however, the cement industry contributes significantly to global carbon dioxide emissions, adversely affecting the ecological balance and raising environmental concerns [4]. Thus, geopolymers have gained prominence [5,6]. Geopolymers are three-dimensional network gels characterized by amorphous and quasicrystalline structures composed of silica–oxygen and aluminum–oxygen tetrahedra [7,8]. The raw materials commonly used to prepare geopolymers include slag, fly ash, calcium carbide slag, and other active components [9]. In recent years, geopolymers have emerged as the ideal solution with which to enhance the performance of foam concrete, potentially replacing cement-based foam concrete and thus aligning with the objectives of green and low-energy building development [10,11]. However, the development and applicability of foamed geopolymer foam concrete (GFC) is still in the early stages. In large-scale production, higher temperatures and alkali concentrations are often required to facilitate geopolymerization reactions, adversely affecting the foam stability and resulting in GFC with poor pore structures or even collapse.

GFC is prepared by producing a foam within a fresh mix, using a foaming agent in an aqueous solution and performing subsequent mixing with a defined ratio of slurry. The natural curing process yields numerous closed micropores. Al_2_O_3_ and SiO_2_ dissociate and re-polymerize during geopolymer activation, which is driven by alkaline raw materials or alkaline substances in the slurry. However, the introduction of alkaline activator gradually destroys the arrangement of active molecules on the surface of the liquid film, increasing the likelihood of bubble coalescence and breakage arising from the increased surface tension as well as decreasing the interface elasticity [12]. Thus, maintaining foam stability is a significant challenge during the reaction process. Synthetic surface-active foaming agents are popular due to their simplicity, cost-effectiveness, and robust foaming performance. Based on their electrical properties, these surfactants can be categorized into anionic, cationic, amphoteric, and non-ionic types [13]. Non-ionic surfactants, including triterpene saponin (TS), are characterized by low-polarity groups that do not exhibit same-charge repulsion, making them prone to close interactions. With its dense hydrophobic groups on the surface of the aqueous solution after dissolution, TS forms a relatively thick molecular film, endowing the bubble wall with high elasticity and strength, rendering it less susceptible to rupture. Importantly, TS foam exhibits remarkable resilience to alkaline conditions, maintaining relative stability within the reaction system, and facilitating the formation of porous structures during the hardening process [14,15,16].

For GFC, the mechanical properties of porous materials are mainly expressed as a function of porosity [17]. It has been shown that porosity, average pore diameter, and average pore surface area influence the mechanical properties of foam concrete to a certain extent [18,19,20,21,22,23,24,25]. Consequently, optimizing the pore structure presents a viable strategy for enhancing the mechanical properties of foams [26,27,28]. For physically foamed GFCs, the interaction between the foaming agent and solid particles significantly influences foam concrete’s pore structure. Different foaming agents have been found to play critical roles in pore structure formation in various systems [29,30,31,32,33,34,35]. If there is a specific interaction between the surfactant head group and a counterion, such as through hydrogen bonding, the distribution of counterions in the stern layer will be closely positioned to the charged head [36]. This phenomenon effectively shields the surface potential, reducing foam drainage and enhancing stability. Consequently, it becomes easier to create more uniform and finer pores using this foamer. However, it is pertinent to note that geopolymer slurries differ from cement-based slurries. The alkali reaction during activation fundamentally alters the ionic distribution of the stern layer, subsequently impacting the stability of the foam, which, in turn, affects pore size distribution, homogeneity, density, and mechanical properties [37]. In order to enhance the pore structure of foam bricks and stabilize the foaming agent, numerous researchers have conducted extensive studies on the formulation of foam agents [38,39,40]. However, these studies do not apply to alkali-supersaturated GFC systems. This study aims to address the issue of foam instability within GFC systems. TS can be foamed at very low concentrations and provide high-quality foam.

In this paper, GFC was prepared using a solid waste-based foam concrete formulation developed in a previous laboratory using fly ash as the primary raw material, carbide slag, and Glauber’s salt for mixing excitation. The formation of the GFC pore structure was explored from the perspective of the foaming agent. GFC was prepared using the anionic surfactant sodium dodecyl sulfate (SDS), the cationic surfactant cetyltrim–thylammonium bromide (CTAB), and the nonionic surfactant TS. The interactions between different foaming agents and geopolymers, as well as their effects on the GFC pore structure formation, were investigated. The underlying reason for the greater stability of the TS foamer compared to other foamers was revealed, and the mechanism through which TS improves the performance of GFC was studied.

## 2. Materials and Methods

### 2.1. Materials

Solid waste-based geopolymer formulations (mix designs) taken from previous laboratory studies were used in this paper [41]. Fly ash (FA) is the main raw material used to form geopolymer networks. This was supplied by China Xinjiang Tianye Co. Carbide slag (CS, generation of Ca(OH)_2_ in this system) and Glauber’s salt (GS, Na_2_SO_4_ ≥ 99%) were used as activators (using calcium hydroxide to react with sodium sulfate to generate sodium hydroxide) for the geopolymer material. The slag (SL) and microsilica slag (MS) were used as auxiliary materials. SL was obtained from Lingshou Jiashuo Building Materials Processing Co., Ltd. (Shijiazhuang, China), while MS was procured from China Xinjiang Tianye Co., Ltd. (Shihezi, China). Then, 10% hollow glass beads (HGB) and 20% cement (CE) were also added to the geopolymer mix to reduce density and increase strength, respectively. HGB was provided by Lingshou County Jashuo Building Materials Co., Ltd., and CE was provided by Xinjiang Tianshan Cement Co. (Urumqi, China).

The CE, FA, CS, and SL were fully ground. The material compositions were tested using an X-ray fluorescence spectrometer (XRF, PANalytical Axios, Alemlo, The Netherlands), and the corresponding results are presented in Table 1. Chemical composition and properties of HGB is presented in Table 2.

**Figure 1:**
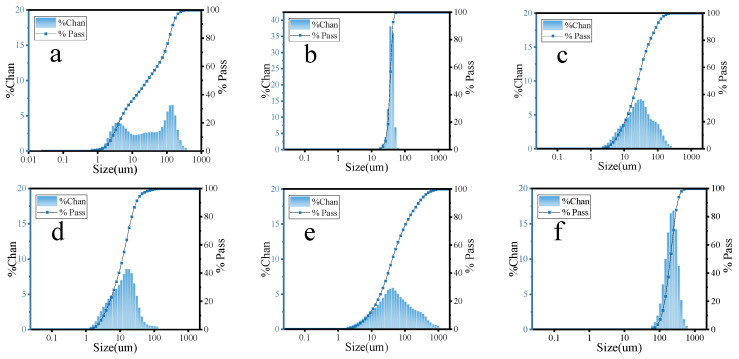
Particle size distribution of (**a**) FA, (**b**) MS, (**c**) CE, (**d**) SL, (**e**) CS, and (**f**) HGB.

The particle sizes of FA, MS, CE, SL, CS, and HGB were tested using a laser particle size tester (Malvern Mastersizer 2000/3000, Malvern, PA, USA) and the results are shown in Figure 1.

Sodium dodecyl sulfate (SDS) and cetyltrimethylammonium bromide (CTAB) with a purity of 95% were obtained from Shanghai McLean Biochemical Science and Technology Co. Ltd. (Shanghai, China). Triterpene saponin (TS) was purchased from Zhengzhou Jenn Pei Chemical Products Co. Ltd. (Zhengzhou, China), and the purity was 98.0%.

### 2.2. Preparation of Precast Foams

**Figure 2:**
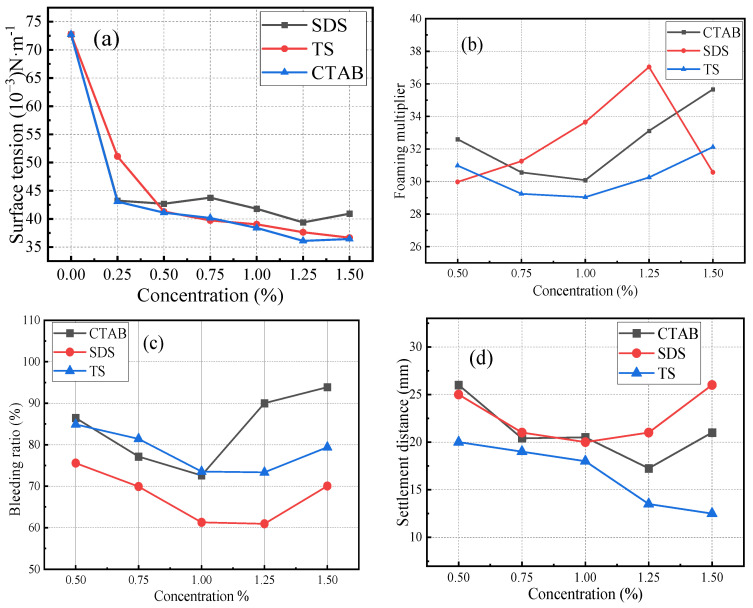
Properties of foaming agents: (**a**) surface tension, (**b**) foaming multiplier, (**c**) bleeding ratio, (**d**) settlement distance. Note: concentration is the mass of surfactant contained per liter of solution.

First, the foaming agent was weighed and mixed with water. It was then magnetically stirred for 15 min to ensure the complete dissolution of the powder. The surface tension of the solution was measured using a maximum bubble pressure surface tension tester. The solution was then foamed using an air compression-type foaming machine. Once the uniform and fine foam flowed out of the outlet, they were transferred into a plastic cylindrical tube with an inner diameter of 60 mm, filling it with foam under its own pressure. The foam volume was adjusted by scraping the tube ends with a steel ruler. The physical properties of the foams were assessed by determining the settling distance and seepage ratio. The surface tension, foaming multiplicity, seepage ratio, and settling distance of different foaming agents are shown in Figure 2. The CMC values were 8.9 × 10^−4^ mol/L for CTAB, 2.4 × 10^−2^ mol/L for SDS, and higher for TS. The foaming ability of TS foamer is not as good as that of the two ionic surfactants. However, the settling distance of TS foam was lower than that of the other two ionic surfactants, and it had high stability and supporting ability. The bleeding ratio of TS was higher than that of SDS. The liquid film of TS was thicker and had a lower water retention capacity than that of SDS.

### 2.3. Preparation of GFC

**Figure 3:**
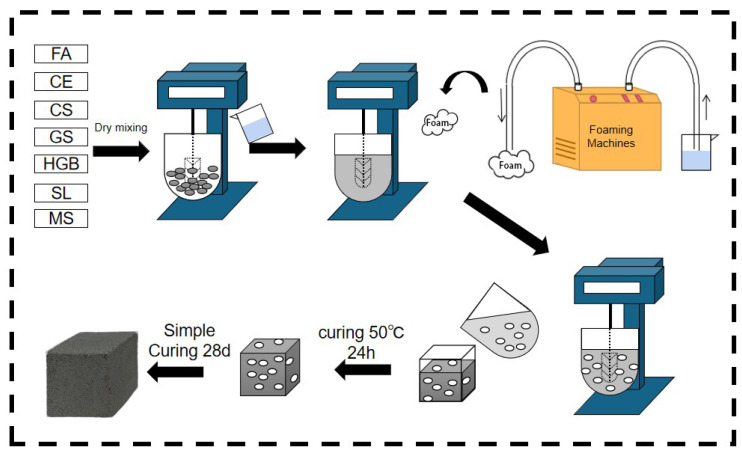
Process flow diagram for preparation of GFC.

The solid waste-based polymer formulations designed in previous laboratory studies were used in this paper [41]. The preparation process of GFC is shown in Figure 3. Raw materials were accurately weighed according to the mix proportions given in Table 3 and mixed with water, following a predefined water–solid ratio. The mixing process involved slow-speed mixing for 30 s, followed by high-speed stirring for an additional 30 s. Subsequently, the freshly prepared foam was added according to Table 4, and slow-speed mixing was continued for 120 s to attain a homogeneous fresh slurry. This fresh slurry was then cast into molds with dimensions of 40 mm × 40 mm × 40 mm and subjected to heat curing at 50 ± 2 °C with a relative humidity of >90% for 24 h, after which demolding took place. Finally, the specimens were transferred to a standard curing box, maintaining a temperature of 20 ± 2 °C and a relative humidity of >90% for an additional 28 days, according to the JC/T 2550-2019 [42]. A Vicat apparatus was used to test the setting time of blank samples. The temperature of the test chamber was 17–25 °C, and the relative humidity was >50%. The temperature of the incubator was 20 ± 1 °C. The initial and final setting times were 300 min and 330 min, respectively.

### 2.4. GFC Test Methods

Flowability was evaluated following the GB/T 8077-2012 [43] standard. The freshly mixed slurry was swiftly poured into a cone measuring 36 × 60 × 60 mm^3^. The top of the cone was leveled, and the cone was removed in 3 s. The maximum diffusion diameter of the slurry on the glass plate was recorded after 30 s.

The dry density of the specimens (40 × 40 × 40 mm^3^) was measured after 28 days of curing following the JC/T 2550-2019 [42] standard. Before measurement, the samples were dried at 105 °C until the weight change of the samples was less than 0.2%. Dry density was calculated using Equation (1).

(1) $$\gamma =\frac{M}{V}\cdot 10^{6}$$

where γ represents the dry density of the sample in kilograms per cubic meter (kg/m^3^), M is the mass of the sample that has been dried to constant weight in grams (g), and V denotes the volume of the sample in cubic millimeters (mm^3^).

The effective foam was calculated according to Equation (2).

(2) $$\eta =\frac{m}{\rho_{1}V_{0}}$$

where η represents the effective foam rate, m is the weight of the slurry and foam, ρ_1_ denotes the density of bricks, and V_0_ is the volume of the slurry and foam.

The compressive strength testing was performed in accordance with JC/T 2550-2019 [42]. We tested 40 mm × 40 mm × 40 mm cubes, cured at a constant quality under controlled conditions of (20 ± 5) °C and (50 ± 15)% relative humidity, at various time intervals (3 days, 7 days, 28 days). Using a uniaxial hydraulic press, a continuous and uniform load was applied at a rate ranging from 0.5 kN/s to 1.0 kN/s until the specimen reached failure. Compressive strength was calculated using Equation (3):

(3) $$\delta_{0}=\frac{P}{A}$$

where δ_0_ represents the compressive strength in megapascals (MPa), P signifies the breaking load in newtons (N), and A is the contact area in square millimeters (mm^2^).

The GFC was sawn from the center and smoothed using sandpaper. Cross-sectional images were taken using a high-definition macro camera. These images were subsequently analyzed using the image processing software Image-J 1.53e to obtain the pore size distribution.

After a 28-day curing period, the samples were immersed in alcohol for 48 h to stop hydration. Subsequently, they were dried and subjected to micromorphological examination using a scanning electron microscope (SEM, Zeiss Gemini 300, Oberkochen, Germany). Thermogravimetric (TG) analysis was also carried out using Netzsch STA 449 F3(Selb, Bavaria, Germany) with a temperature range of 30–1000 °C and a ramp rate of 10 °C/min.

## 3. Results and Discussion

### 3.1. Fluidity

**Figure 4:**
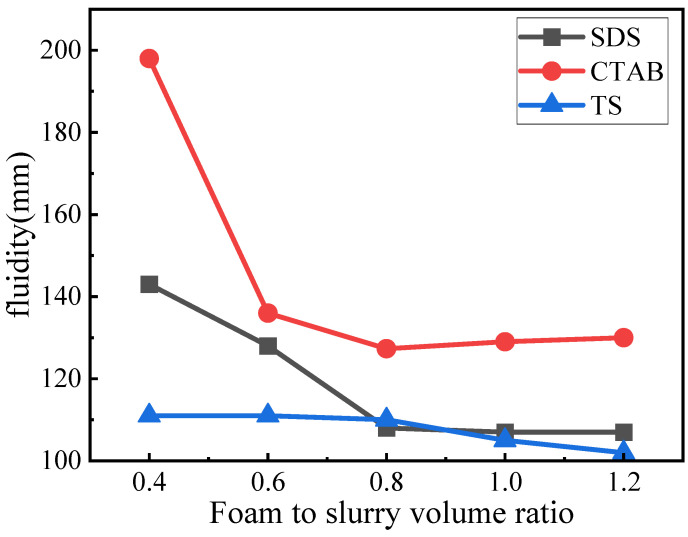
Fluidity of GFC fresh slurry.

Fluidity indicates the viscosity of the fresh slurry and significantly impacts the microstructure of GFC. The fluidity of the GFC fresh slurry is shown in Figure 4. The fluidity was gradually reduced with the addition of foaming admixtures. However, the corresponding trend for each is significantly different. For SDS, the fluidity decreases with the increase in concentration; however, when the SDS concentration is >0.8, the corresponding decline in fluidity slows down to be in a more stable state. When the foam content is less, the flow is higher, and the wrapping force of the slurry for the foam is smaller, which can easily rupture the foam and lead to a further increase in fluidity. The fluidity of GFC with CTAB addition is overall larger. CTAB-0.4 Foam rupture is severe. With an increase in CTAB foam content of 0.6, the volume and slurry pressure around the foam increased while the fluidity decreased significantly. Simultaneously, although the tendency of foam rupture was reduced, there remained an obvious fusion phenomenon compared to the other two foams, with fluidity decreasing to a minimum of 130 mm. TS foams had the most stable fluidity, showing only a slight decrease in fluidity with increasing volume. Compared to the other two electrophilic foams, TS foams showed no significant foam rupture or fusion, suggesting a potential interaction between TS foam and geopolymer slurry that enhances stability.

### 3.2. Dry Apparent Density

**Figure 5:**
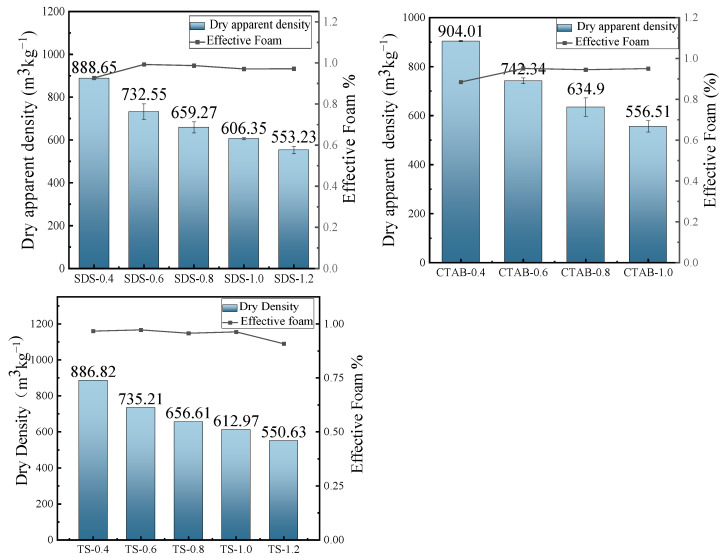
Dry apparent density and effective foam.

The dry density of GFC changes with the addition of foam, as shown in Figure 5. The foam introduced by the foaming agent significantly increases the total porosity, reducing the resulting density of the specimens. The variation in fluidity gives rise to changes in the bubble constraint force of the slurry to the foam. The fluidity increases at a reduced foam content, resulting in a bubble constraint force between the slurry and the foam. CTAB and SDS are unstable in the slurry at a lower foam content, with a slight decrease in effective foam and greater density. The effective foam of TS slightly decreases when the foam content is higher. However, CTAB-1.2 experiences mold collapse because the stabilization time of the foam in the slurry is shorter than the hardening (setting) time of the slurry. The presence of insufficiently bound foam in the slurry, occurring due to low force, results in the destruction or disintegration of the foam.

### 3.3. Porosity and Pore Structure

**Figure 6:**
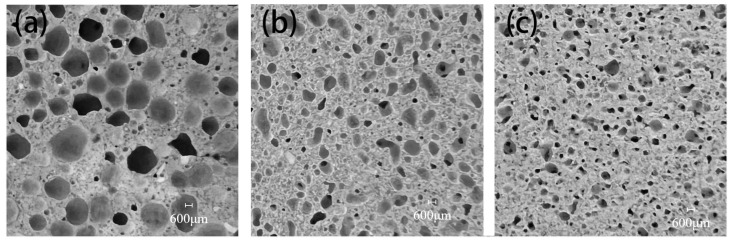
Cross-section of (**a**) CTAB-0.8, (**b**) SDS-0.8, and (**c**) TS-0.8.

The pore structures of CTAB-0.8, SDS-0.8, and TS-0.8 are shown in Figure 6. It can be seen that CTAB-0.8 has a larger pore diameter, whereas SDS-0.8 and TS-0.8 exhibit relatively smaller pore diameters. In the unhardened phase of the GFC, the liquid film of the foam is encapsulated by the ground polymer slurry, and the foam is subject to binding forces from the slurry. At a foam content ratio of 0.8, CTAB-0.8 demonstrates the highest flowability, and the ground polymer slurry has a lower binding force on the foam, leading to noticeable foam merging and the formation of larger pores. Conversely, TS-0.8 has the lowest fluidity, resulting in a stronger binding force from the slurry on the foam, making it difficult for the foam to split and merge, thus forming smaller pores.

**Figure 7:**
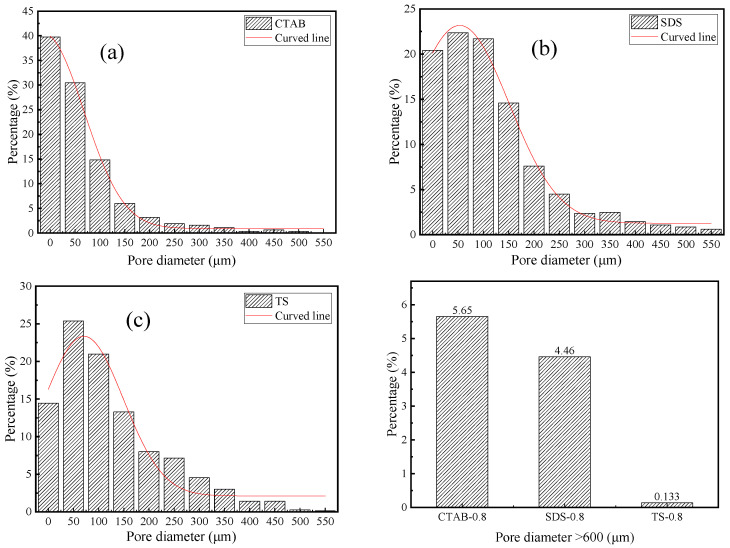
Pore diameter distribution of (**a**) CTAB-0.8, (**b**) SDS-0.8, and (**c**) TS-0.8.

The pore size distribution of SDS, CTAB, and TS is shown in Figure 7. CTAB-0.8 has a higher percentage of pores with diameters less than 50 μm and greater than 600 μm, indicating a non-uniform pore structure distribution and significant foam merging and splitting issues in the unhardened stage of GFC. In contrast, TS-0.8 exhibits a more uniform pore distribution, with pores greater than 600 μm occupying less space. These large pores are the main reason for the reduced strength [44]. TS-0.8 has the fewest pores larger than 600 μm, accounting for only 0.13% of the total, indicating that TS is most stable in the GFC environment, with TS foams being less likely to merge or split during the GFC-hardening stage. The air voids entrapped by the cationic surfactant were much coarser. The pores entrapped by anionic surfactant SDS and nonionic TS surfactant were medium and small in size, respectively, which is consistent with the previous findings in terms of cement-based foam concrete [45].

**Figure 8:**
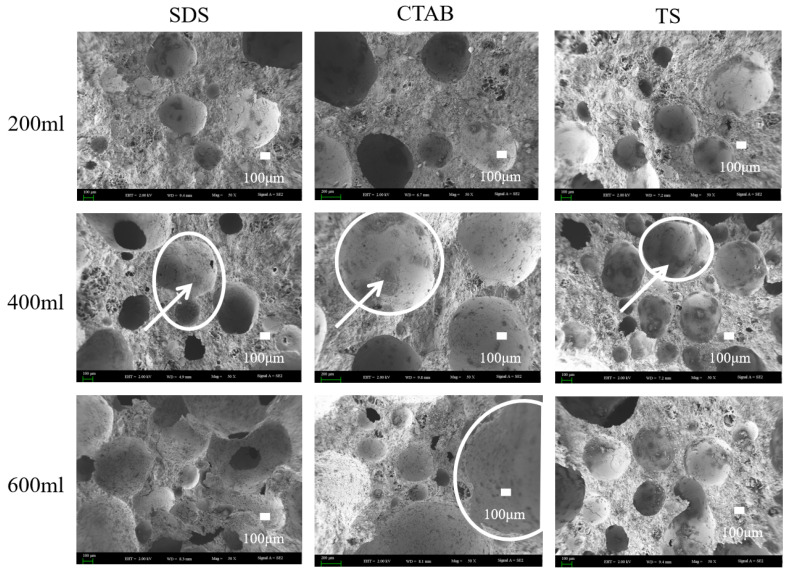
SEM images of GFC microstructure.

Scanning electron microscopy (SEM) imaging was carried out on GFC specimens with foam-to-solid ratios of 0.4, 0.8, and 1.2, further clarifying the foaming mechanism (merge and split) in fresh geopolymers. The SEM images are shown in Figure 8. Circles show pores and arrows show hydration products of the pores. When the foam content is less, the bubbles are filled with more gelling material between the bubbles, and the chances of foam-to-foam contact are slim. Thus, the foam is better dispersed, and pore tortuosity is higher. When the foam-to-solid ratio increases to 0.8, the foam changes due to varying electrical properties. The pore size of SDS-0.8 is relatively small. However, the pore connectivity is pronounced, which is attributed to the distance between the SDS foams being close in the unhardened stage of the GFC. The water film disappears upon hardening, and connecting pores are generated between closely situated foam particles. CTAB-0.8 exhibits larger round holes, with some tiny holes sandwiched around the walls. This may be attributed to the instability of CTAB foam in GFC, which promotes the merging of bubbles into larger entities. TS-0.8 did not exhibit foam fusion and splitting phenomena and entrapped some smaller pores, indicating that the TS foam could stably exist in geopolymer slurry. When the ratio of foam to slurry was increased to 1.2, the bubble behavior became more prominent. SDS-1.2 showed larger bubbles, and the bubble fusion phenomenon became more pronounced while the through pores also increased. The pores of CTAB-1.2 became larger, and more fine pores were also observed. The pore deformation phenomenon noted in TS-1.2 could also be seen. Still, the pores were generally spherical and had a more uniform distribution, which was different from the conclusions reached for cementitious materials. In cement-based foam concrete, anionic surfactants produce intact circular pores, whereas nonionic and cationic surfactants tend to produce connected pores [30]. Variations in the liquid environment in which the foam resides and the interaction forces with solid particles contribute to this disparity.

### 3.4. Compressive Strength

**Figure 9:**
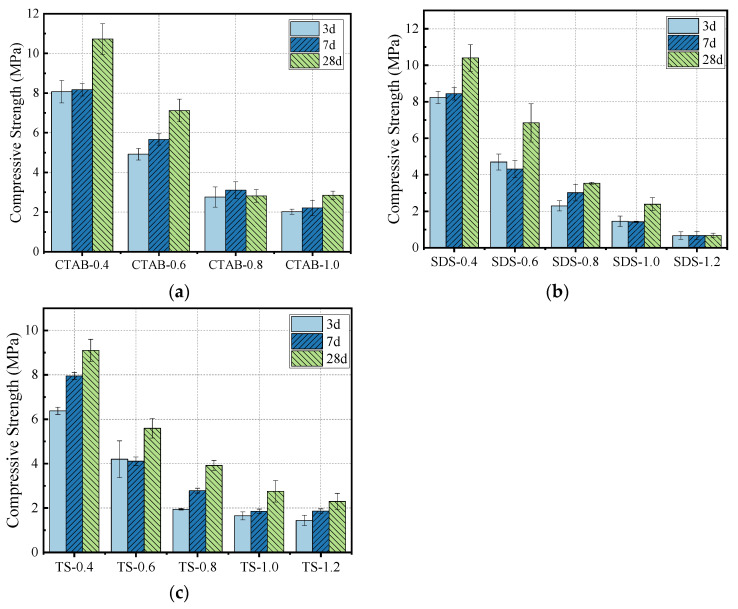
Compressive strength of (**a**) CTAB-0.8, (**b**) SDS-0.8, and (**c**) TS-0.8.

The effect of foam content on compressive strength is shown in Figure 9. A decrease in density leads to a decrease in the corresponding compressive strength, typically observed for various cement-based composites. As the curing duration increases, the geopolymerization of GFC continues, increasing the compressive strength. After adding the foam, the density decreases, which lowers the resulting compressive strength. The decrease in compressive strength with CTAB addition was the most significant, followed by SDS and TS. At a foam-to-solid volumetric ratio of 0.8 and a specimen density of 700 kg/m^3^, the compressive strength of TS-0.8 was higher than that of SDS-0.8 and CTAB-0.8 by 10.78% and 28.13%, respectively. The change in compressive strength correlates with the pore structure of GFC. GFC produced using CTAB foam has larger pores and lower compressive strength. In contrast, GFC produced with SDS foam has more interconnected holes, while GFC with TS foam shows a uniform distribution with fewer through holes, thus resulting in higher compressive strength. Further, GFC hydration also affects strength development.

### 3.5. Hydration Analysis

**Figure 10:**
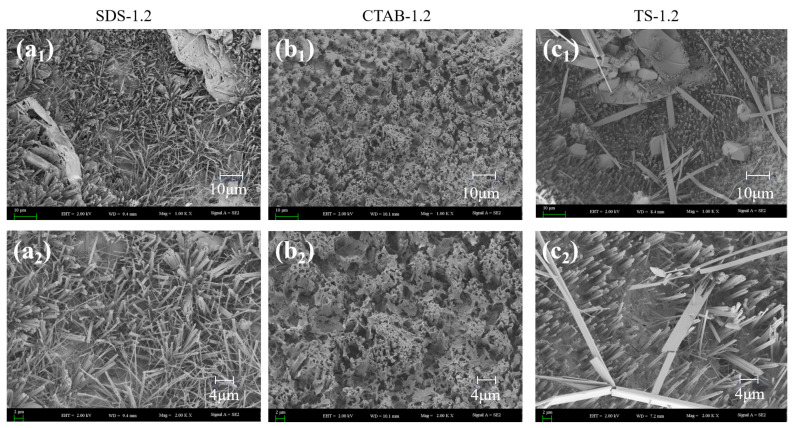
SEM images of the hydration products of (**a_1_**,**a_2_**) SDS-1.2, (**b_1_**,**b_2_**) CTAB-1.2, and (**c_1_**,**c_2_**) TS-1.2.

In addition to pore distribution affecting the compressive strength of the GFC, the firmness of the foam wall affects the compressive strength of the GFC. The hardness of the foam wall is related to the degree of hydration and geopolymerization. Due to the different electrical properties of the foaming agents, there are different interactions with the solid particles of the gelling material, affecting the degree of hydration of the ground polymer. SEM images of the foam geopolymers prepared with the three foaming agents are shown in Figure 10. In Figure 10, there are many needle/rod-like ettringite (AFt) crystals and amorphous C-(A)-S-H gels [46]. Due to the disparity in electrical properties, the morphologies of the hydration products were significantly different. Irregularly distributed needle-like hydrated gels were formed on the pore surface of the GFC prepared with the anionic surfactant SDS, whereas the cationic surfactant CTAB generated honeycomb-like hydrated gels and the non-ionic surfactant TS generated longitudinally distributed hydrated gels.

**Figure 11:**
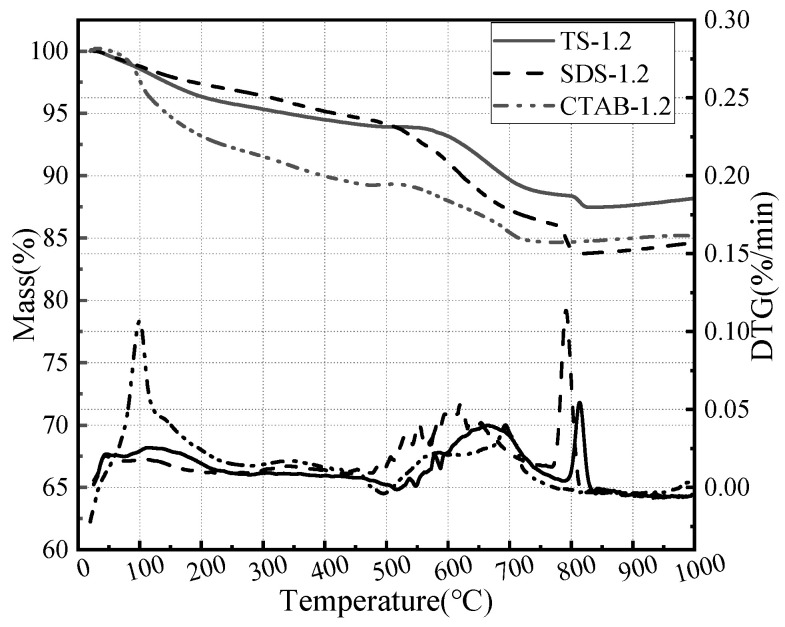
Thermogravimetric analysis of SDS-1.2, CTAB-1.2, and TS-1.2.

In order to investigate the effect of foaming agents on GFC hydration, thermogravimetric analyses were carried out on a foam geopolymer prepared by three foaming agents. The corresponding results are shown in Figure 11. The decomposition of GFC can be divided into three stages based on temperature ranges: the evaporation of water and hydrate from 30 to 400 °C, followed by silicate decomposition and portlandite dehydrogenation from 400 to 600 °C, and finally calcite decomposition from 600 to 800 °C [47,48]. The honeycomb structure of CTAB hydration products retains more free water. SDS and TS exhibit silicate and calcite decomposition. The foaming agent affects the hydration process of GFC and the morphology of the hydration products.

### 3.6. Mechanism Analysis

**Figure 12:**
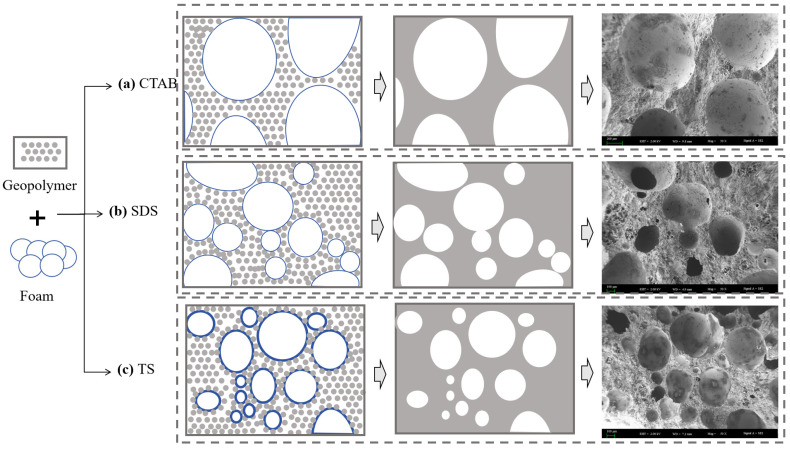
Pore evolution of GFC.

The behaviors of three foams within the GFC slurry are shown in Figure 12. Cationic surfactants do not strongly interact with the geopolymer matrix. The geopolymer slurry has less friction with the foam, resulting in easier consolidation of the foam. Eventually, large-sized pores are formed. SDS has a certain mutual attraction with geopolymer particles and can adsorb particles on the surface [45]. However, the liquid film of the SDS foam is thin, rendering it susceptible to the formation of interconnected pores at points where the foams make contact with each other. Mutual attraction exists between non-ionic TS particles and geopolymer matrix [16]. At the same time, hydrogen bonding adsorbs a thicker layer of water so that the foams do not merge easily with each other and there are few connected pores [49]. The use of TS foaming agents enables a more homogeneous, more compact, and denser microstructure, facilitating improvements to the mechanical strength of GFC.

**Figure 13:**
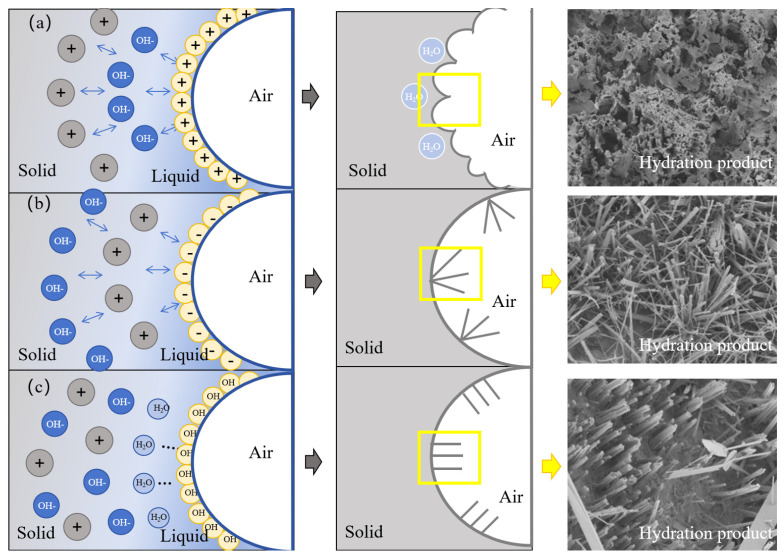
Hydration mechanism of (**a**) CTAB, (**b**) SDS, and (**c**) TS.

The hydration process of GFC prepared by different foaming agents is illustrated in Figure 13. The yellow box shows the hydration product morphology. When the GFC matrix is fresh and not yet hardened, the air in the foam evolves into the pores, and the liquid film of the foam evolves into the outer walls of the pores. Subsequently, the geopolymer starts undergoing hydration reactions. The formation of the hydration products attached to the surface of the pore wall is affected by the influence of the surfactant and exhibits different morphologies [50]. CTAB cationic foams first adsorb OH^-^ from the environment, followed by cations such as Si^2+^, Al^3+^ and Ca^2+^. Charged particles in the same layer repel one another due to carrying the same charge. As a result, the hydration products grow in different directions and show an uneven arrangement. At the same time, a water-rich region exists at the beginning of the geopolymer hydration reaction. Since the cationic foam first attracts OH^-^ rather than cations (such as Si^2+^, Al^3+^ and Ca^2+^), the cations are hydrated towards the interior of the water-rich region. The water-rich region disappears when the reaction is complete, leaving a honeycomb of uneven hydration products. At the same time, a portion of free water is encapsulated inside. The SDS anionic foam first adsorbs cations such as Si^2+^, Al^3+^ and Ca^2+^. These cations produce electrostatic repulsion on the surface, leading to the irregular growth of hydration products on the surface. Eventually, irregularly distributed hydration products are formed [51]. TS foams are attracted to water molecules through hydroxyl groups. Si^2+^, Al^3+^, and Ca^2+^ are evenly dispersed in the liquid phase with OH^-^ without electrostatic repulsion. As a result, uniformly distributed needle/columnar hydration products are produced [52].

## 4. Conclusions

In this study, the application of triterpene saponins (TS) as a foaming agent in geopolymer foam concrete (GFC) is explored. The effects of three electrically charged foaming agents on GFC fluidity, compressive strength, pore structure, and hydration products were studied. The following conclusions are drawn from the obtained results.

(1) The choice of foaming agent significantly affects the performance of GFC, especially in its freshly mixed state. Notably, GFC prepared with TS foaming agent demonstrates the highest stability.
(2) Foaming agents affect the hydration process of GFC, and the morphology of the hydration products formed varies considerably. TS shows a better water retention effect due to hydrogen bonding, promoting the hydration process.
(3) The porosity and pore structure are also significantly influenced by the foaming agents. If the foam is poorly stabilized, it cannot resist the destructive effects of the GFC alkali reaction process, and thus, the pore connectivity is high and the pore structure is poor. TS is highly stable in the GFC matrix, so the corresponding pore size is small, and the connectivity is low in the hardened GFC.

The findings of this study confirm the suitability of TS foaming admixtures for producing GFC. TS shows excellent stability under salt supersaturation and alkaline environments in GFC paste. This study provides innovative approaches for developing environmentally friendly, solid waste-based building composites.

## Figures and Tables

**Table 1:** The chemical composition of FA, CE, SL, and CS.

Material	SiO_2_	Al_2_O_3_	CaO	Fe_2_O_3_	MgO	NaO	SO_3_	Other	Loss on Ignition
FA	41.43	18.90	15.10	10.25	4.28	4.13	2.43	3.48	5.40
CE	18.71	3.93	64.77	4.24	1.77	0.90	3.32	2.36	5.09
SL	27.51	11.93	47.64	0.40	7.38	0.47	2.55	2.12	5.34
CS	4.62	1.87	91.41	0.38	-	-	0.90	0.82	1.67

**Table 2:** Chemical composition and properties of HGB.

SiO_2_	Al_2_O_3_	K_2_O	Fe_2_O_3_	Other	Flotation Rate (%)	Thermal Conductivity W/(m·k)	Density (kg·m^−3^)
58.80	28.72	3.60	2.40	6.48	80.00	0.05	360

**Table 3:** Geopolymer mix proportions.

FA (g)	CE (g)	SL (g)	CS (g)	GS (g)	MS (g)	HGB (g)	W/S
285	120	60	30	30	15	60	0.45

Note: W/S stands for water–solid ratio. The amounts of W/S were calculated according to the mass of the matrix.

**Table 4:** Mix proportions of foamed geopolymer composites.

Mix	Target Density (kg/m^3^)	Foaming Agent	Geopolymer (g)	Foam (mL)	Actual Average Density (kg/m^3^)
SDS-0.4	850	SDS	600	200	888.6
SDS-0.6	750	SDS	600	300	742.6
SDS-0.8	650	SDS	600	400	659.3
SDS-1	600	SDS	600	500	606.4
SDS-1.2	550	SDS	600	600	553.2
CTAB-0.4	850	CTAB	600	200	904.0
CTAB-0.6	750	CTAB	600	300	742.3
CTAB-0.8	650	CTAB	600	400	634.9
CTAB-1	600	CTAB	600	500	556.5
CTAB-1.2	550	CTAB	600	600	-
TS-0.4	850	TS	600	200	886.8
TS-0.6	750	TS	600	300	735.2
TS-0.8	650	TS	600	400	656.6
TS-1	600	TS	600	500	612.9
TS-1.2	550	TS	600	600	550.6

## Data Availability

Data are contained within the article.
